# Value of 4D CT Angiography Combined with Whole Brain CT Perfusion Imaging Feature Analysis under Deep Learning in Imaging Examination of Acute Ischemic Stroke

**DOI:** 10.1155/2022/2286413

**Published:** 2022-06-13

**Authors:** Jingshan Tao, Yong Cai, Yisheng Dai, Yingdi Xie, Hailing Liu, Xiaojin Zang

**Affiliations:** ^1^Department of Radiology, Binhai County People's Hospital, Yancheng 224500, Jiangsu, China; ^2^Department of Neurology, Binhai County People's Hospital, Yancheng 224500, Jiangsu, China

## Abstract

This study was aimed at investigating the application of deep learning 4D computed tomography angiography (CTA) combined with whole brain CT perfusion (CTP) imaging in acute ischemic stroke (AIS). A total of 46 patients with ischemic stroke were selected from the hospital as the research objects. Image quality was analyzed after the 4D CTA images were obtained by perfusion imaging. The results showed that whole brain perfusion imaging based on FCN can achieve automatic segmentation. FCN segmentation results took a short time, an average of 2-3 seconds, and the Dice similarity coefficient (DSC) and mean absolute distance (MAD) were lower than those of other algorithms. FCN segmentation distance was 17.87. The parameters of the central area, the peripheral area, and the mirror area of the perfusion map were compared, and the mean transit time (MTT) and time to peak (TTP) of the lesion were prolonged compared with the mirror area. Moreover, the peripheral CBV was increased, and the differences between the parameters were significant (*P* < 0.05). In conclusion, using the deep learning FCN network, 4D CTA combined with whole brain CTP imaging technology can effectively analyze the perfusion state and achieve clinically personalized treatment.

## 1. Introduction

Stroke is a sudden disease with high disability rate [1, 2]. Stroke stenosis is the most common cause of ischemic stroke [3, 4]. Imaging examination of acute ischemic stroke (AIS) is classified into neuroimaging and advanced neuroimaging [5–7]. 4D computed tomography angiography (CTA) can be enhanced with contrast agent images. Compared with conventional CTA, 4D CTA imaging shows the changes of cerebral blood vessels within a certain period, accurately showing the dynamic changes of blood vessels and blood flow. CTA combined with CT perfusion (CTP) can evaluate ischemia, and perfusion imaging plays an important role in predicting the efficacy of intravenous thrombolytic therapy [8–10]. Whole brain CTP is a functional imaging technology, which is also of great value for AIS. Functional maps and cerebral perfusion parameters obtained by different digital models can be used to find the changes of blood perfusion volume [11, 12]. CTP imaging is widely used in patients with ultra-early AIS. Abnormal perfusion areas can be found in routine examination, and infarct core and ischemic penumbra volume can be evaluated, which is conducive to the individualized treatment of thrombolytic therapy [13, 14]. 4D CTA combined with whole brain CTP can greatly improve the scanning rate, more comprehensive display of the lesion range, greatly reduce the rate of missed diagnosis, and improve the time resolution of examination. Deep learning-based whole brain perfusion imaging can achieve more treatment time for patients [15, 16].

With its fast nonlinear mapping ability and ultra-fast propagation ability, it has been widely applied in signal processing and many fields. Its application in image recognition, information processing, model identification, and other aspects is also quite good, and the operation of training depth model becomes more convenient [17]. With the application of artificial intelligence in computers, deep learning has been widely used in organ segmentation and lesion detection in images [18, 19]. Convolutional neural network (CNN) is widely used, but translation invariance and pooling layer exist, which will affect the final model performance. Fully convolutional network (FCN) replaces the full connection layer behind the traditional convolutional network with the convolution layer, which can solve the impact of convolution and pooling on the image size. FCN classifies images at the pixel level, can solve the problem of semantic image segmentation, and can also receive input images of any size. Upsampling with deconvolution layer can restore the size of the input, retain the spatial information in the original input image, and finally classify each pixel on the feature map of upsampling.

In this study, the CNN model of deep learning was converted into the fully convolutional neural network (FCN) of image semantic segmentation to automatically extract the deep features of image data of AIS patients and realize automatic segmentation of perfusion images. Using simulated datasets in deep learning neural network, 4D CTA and CTP realize “one-stop” examination to provide more comprehensive and detailed imaging information for AIS patients. Combining cerebrovascular morphological changes and hemodynamic changes, the characteristics of AIS patients were analyzed from different perspectives to provide effective treatment for patients, so as to provide reference for clinical diagnosis of AIS patients.

## 2. Materials and Methods

### 2.1. Patient Information

Forty-six suspected ischemic stroke patients in the hospital from June 2019 to June 2021 were selected as the research objects. Among them, 28 were male and 18 were female. Patients were 42–70 years old, with an average age of 63.62 ± 7.14 years. All patients underwent one-stop dynamic volume CTA-CTP examination, and six patients were admitted because of dizziness. There were 12 patients in 24 ~ 48 hours after onset. No clinical treatment was performed before CTA-CTP examination. There were 15 patients with ischemic stroke 6–14 days after onset. Clinical treatment, such as anticoagulation and fibrinogen reduction, was performed before CTA-CTP examination. Thirteen of the patients were acute stroke patients within four to five hours and underwent thrombolytic therapy with 1 mg/kg heavy tissue fibrinolytic activator. This experiment was approved by ethics committee of the hospital, and all relevant personnel signed the informed consent form.

The patients met the *North American Symptomatic Carotid Endarterectomy Trial (NASCET)*. The stenosis of middle cerebral artery (MCA) or internal carotid artery (ICA) was graded into mild (0–29%), moderate (23–69%), severe (70%–99%), and occlusion (100%).

Inclusion criteria were as follows: (I) clinical diagnosis of acute cerebral hemorrhage; (II) complete clinical data; (III) those who cooperated with medical workers independently; (IV) those who had not interrupted the treatment in this hospital; and (V) those who met the treatment indications.

Exclusion criteria were as follows: (I) patients combined with no serious heart and kidney disease and iodine contrast agent allergy; (II) patients with mental diseases; (III) patients with poor treatment compliance; (IV) patients with communication disorder; (V) discontinuation of treatment due to multiple reasons; and (VI) patients with cerebral hemorrhage and other lesions.

### 2.2. Whole Brain Perfusion Imaging

Patients were examined by GE660 functional 128-slice CT equipment. Cranial carotid artery CT data were obtained. The total scanning range was 30 cm, and the tube transfer time was 0.5 s/r, covering the whole brain. Scanning conditions: 80 kV, 300 mA. Layer thickness was 1.25 mm, matrix was 512 × 512, and scanning field was 200 mm × 200 mm. The scanning interval was 1.5 s, and a total of 22 scans were performed. 45.14 s was the total scanning time. Intravenous injection of nonionic contrast media included normal saline (30 mL) and iopromide (370 mg/mL, 30 mL). The injection flow rate was 5 mL/s, and the dynamic scanning delay was 5 s. The original data were reconstructed and analyzed by dynamic CTA perfusion imaging.

After 8–10 minutes of CTP scanning, the contrast agent iopromide 50 mL was injected, and automatic tracking triggered scanning was started after 8 s delay. Tube current was 250 mAs, voltage was 120 kV, and the layer thickness was 1.25 mm.

**Figure 1:**
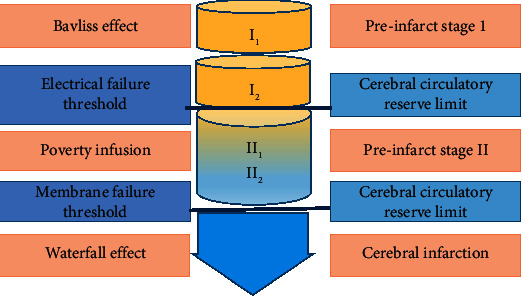
Schematic diagram of imaging staging of regional cerebral microcirculation disorders in the precerebral infarction period.

Figure 1 shows the imaging staging of regional cerebral microcirculation disorders in the precerebral infarction stage. The CTP manifestations before cerebral infarction are shown in Table 1.

### 2.3. Image Processing

The original data are transmitted to Vitrea workstation for processing through data packets. The perfusion parameters were automatically generated by the perfusion software, including mean transit time (MTT), cerebral blood volume (CBV), cerebral blood flow (CBF), time to peak (TTP), and delay time. The perfusion parameter images were composed of 420 images with a thickness of 0.5 mm. 4D CTA images and perfusion parameters received any plane reconstruction, and then quantitative analysis of perfusion parameters was implemented. The collateral blood flow state was evaluated according to the reconstruction degree of distal occluded vessels in reconstructed images, which can be “good” or “reduced.” The “good” condition was that the distal branch vessels of MCA or ICA occlusion were no less than 50%. The “reduced” lateral branches showed that MCA or ICA occlusion of distal branches was less than 50%. The images were obtained by advanced vascular analysis software, and the compensations of arterial and collateral vessels were observed by two neuroimaging physicians.

### 2.4. Network Structure Model

**Figure 2:**
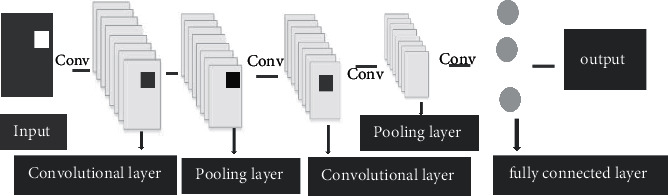
Schematic diagram of a typical CNN structure.

The single use of traditional CNN for image segmentation tasks in deep learning is not suitable for the prediction of target objects. When CNN solved the image segmentation problem, some scholars proposed to divide patch into several small images. The category of image block is the category to which the center pixel of each image block belongs. The image is matched according to the judged category and then used as the input information of the network. In deep learning network, pixel information of different scales can be combined to extract the best size information. The size of convolution kernel can improve the running speed of neural network. Deconvolution network can be used to solve the problem of image semantic segmentation and can also be used to visualize the features extracted by CNN.  Figure 2 shows a schematic diagram of a typical CNN. Inspired by the local perception of biological vision, local receptive fields, pooling, and weight sharing are integrated into the network, which can make the network have stronger feature extraction ability, considerably reduce the content of network parameters, and effectively avoid the occurrence of overfitting in the training process. Typical CNN includes input layer, convolution layer, and full connection layer. The core layer is convolution layer. Pooling can reduce the dimension of features, and the convolution needs nonlinear transformation after operation. The full connection layer transforms the matrix features into column vectors for the connection of classifiers.

The feature map obtained from the original image in the CNN model is used as the input of the convolution layer. After the operation, the new feature map enters the convolution operation of the next layer. In general, for continuous functions, two functions are required for integral operation. If there are *g*(*x*) and *f* (*x*) in *M* which are two continuous integrable functions, the convolution definition equation is expressed as follows.

(1) $$\begin{array}{c} w\left(t\right)=\int_{℘=-\infty }^{+\infty }+f\left(℘\right)g\left(t-℘\right)\text{d}℘. \end{array}$$

If both *g*(*x*) and *f* (*x*) are discrete variables, the convolution operation is transformed from the integral operation of the continuous function to the corresponding summation operation. The convolution expression equation is expressed as follows.

(2) $$\begin{array}{c} w\left(t\right)=\int_{℘=-\infty }^{+\infty }+f\left(℘\right)g\left(t-℘\right). \end{array}$$

In the CNN calculation process, the feature map of the input layer and other layers is a high-dimensional array, the two-dimensional tensor is called a two-dimensional matrix, and the three-dimensional tensor is called a three-dimensional matrix, which is represented by the form (*X*, *Y*, *Z*). The number of channels in an image is represented by *X*. If the color image *X* value is 3, the gray image *C* is 1, and *Y* and *Z* represent the height and width of the image matrix, respectively, the expression in the convolution calculation is as follows.

(3) $$\begin{array}{c} U_{j}^{l}=\sum_{i\in C_{\text{in}}}x_{i}^{l-1}⊗s_{ij}^{l}+b_{j}^{l},\\ X^{l}=f\left(U^{l}\right). \end{array}$$

In the above equations, *x*_*j*_^*l*^ is the output of the previous layer *l* − 1 layer, *s*_*ij*_^*l*^ is the connection parameter between any neuron of the *i*-th layer of the *l* − 1 layer and the *l*-th layer, and *U*_*j*_^*l*^ is the operation result obtained by any *j*-th neuron in the *l*-th layer of the network.

The sigmoid function is used as the activation function, and the expression of the sigmoid function is as follows.

(4) $$\begin{array}{c} f\left(j_{k}^{l}\right)=\frac{1}{1+\exp\left(-j_{k}^{l}\right)}=\frac{1}{1+\exp\left(-A_{p}^{l-1}*c_{k}^{l}\right)}. \end{array}$$

The pooling layer extracts its maximum value as a feature value in a local range based on the operation of the convolution layer. It is only necessary to perform nonlinear operations on the mapped image, and then the feature map size of the image is expressed as follows.

(5) $$\begin{array}{c} \frac{\left(\alpha^{l-1}-m^{l}+1\right)}{s}\times \frac{\left(\alpha^{l-1}-m^{l}+1\right)}{s}. \end{array}$$

In the above equation, *s* is the size of the pooling operation.

For classified CNN algorithms, the output layer should be a classifier, and the softmax classifier (SMC) is one of the commonly used classifiers in CNNs. SMC is a supervised logistic regression model, which is calculated as follows.

(6) $$\begin{array}{c} B_{C^{l}}^{l}\left(y^{l-1}\right)=\frac{1}{1+\exp\left(-C^{l}y^{l-1}\right)}. \end{array}$$

According to the logistic regression cost function, the cost function of softmax regression can be obtained as follows.

(7) $$\begin{array}{c} J\left(C^{l}\right)=-\frac{1}{N}\left\{\sum_{i=1}^{N}C\left(i\right)\log\;C^{l}\left[y\left(i\right)\right]+\left[1-y\left(i\right)\right]\log\left(1-C^{l}\right)y\left(i\right)\right\}. \end{array}$$

In the above equation, *y*(*i*) represents the training set, and *y*(*i*) ∈ {1,2,…, *k*}.

For each image block, the category of the image block finally obtained through the sliding window is expressed as follows.

(8) $$\begin{array}{c} i=\arg\max\left[p_{C^{l}}^{l}\left(y^{l-1}\right)\right]. \end{array}$$

In the above equation, *p*_*C*^*l*^_^*l*^ represents the probability that the image block *x* is classified as *j*, and its calculation method is as follows.

(9) $$\begin{array}{c} p_{C^{l}}^{l}=\frac{e^{\theta_{j}^{r}\left|l\right|}}{\sum_{i=1}^{k}e^{\theta_{j}^{r}x^{l}}}. \end{array}$$

In the above equation, *θ* is the model parameter.

(10) $$\begin{array}{c} \frac{1}{\sum_{i=1}^{k}e^{\theta_{j}^{r}x^{l}}}. \end{array}$$

Equation (10) is the probability distribution normalization term.

It is assumed that the kernel convolution area of the feature map *A* is *Q*_*i*_, and *O* is the offset of a specific position *i* on *Q*_*i*_; then, the image position after the conventional convolution operation in the deformable branch can be expressed as follows.

(11) $$\begin{array}{c} Q_{i}=Q+A,\\ N_{i}=Q_{i}+O=Q+A+O. \end{array}$$

In the above equations, *N*_*i*_ represents the new coordinate value of the image *A* after the convolution operation.

Based on region segmentation performance evaluation, Dice similarity coefficient (DSC) is used for evaluation. The DSC is often used to evaluate the similarity between the automatic image segmentation results and the artificial results. The larger the value, the better the segmentation effect. The calculation for the DSC is as follows.

(12) $$\begin{array}{c} \text{Dice}\left(A,B\right)=\frac{2\left|A\cap B\right|}{\left|A\right|+\left|B\right|}. \end{array}$$

In the above equation, *A* is the pixel set for automatically segmenting the image, *B* is the pixel set for manually delineating the image, and *C* is the number of all pixels in the image.

### 2.5. Experimental Configuration and Process

**Figure 3:**
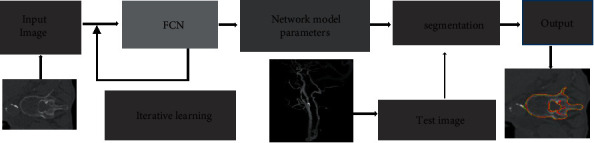
Flowchart of image processing.

Medical Image Computing and Computer Assisted Intervention 2016 dataset was used for training on the network catenary. 1542 images of the training set and 900 images of the test set were randomly selected from the dataset. The size of the input images was 521 pixels *∗* 512*∗*. The research was carried out in Ubuntu system, and the constructed FCN and deconvolution network were run on GPU (Intel I7-7700) with 16 GB memory and NVIDIA GTX1070 GPU configuration. The neural network used in the experiment converged after continuous forward propagation and backpropagation iterative learning, and the parameters were saved. In the test stage, only the test image was input, and the segmentation result was obtained through the discrimination and classification of each pixel by softmax classification layer (Figure 3). In this study, different parameter values were selected to test the sensitivity, and the accuracy of different parameters on the whole image segmentation was analyzed.

### 2.6. Statistical Analysis

SPSS 19.0 was used for data processing in this study. Measurement data that conformed to normal distribution were expressed as mean ± standard deviation ($\bar{x}$ ± *s*), and the count data were expressed by frequency or percentage (%). Data differences were compared using the *t* test, and the chi-square test was used for quality comparison. *P* < 0.05 was considered statistically significant; otherwise, it was insignificant.

## 3. Results

### 3.1. Network Parameter Sensitivity Test

**Figure 4:**
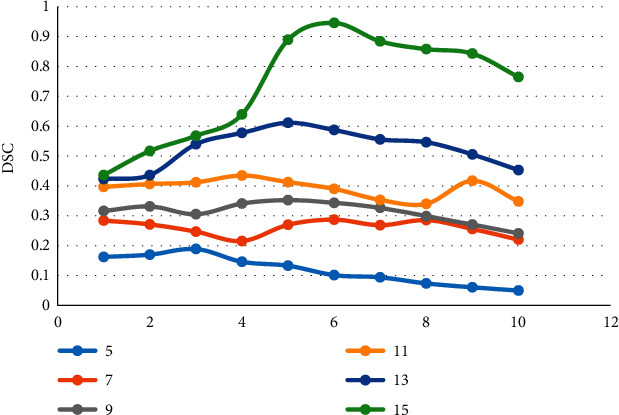
Sensitivity test.

The convolution layer of the network model replaced the 5 *∗* 5 large kernel convolution of the single layer of the network with two consecutive 3 *∗* 3 convolutions without changing the size of the extracted feature map. Multiple convolution operations also included nonlinear capabilities of multiple activation functions. In the selection of parameters, different parameter settings also had a certain image on the segmentation effect. The overall fluctuation effect was small, and the difference value set within a certain range had little effect on the overall segmentation accuracy of the image. The values of *θ* were 5, 7, 9, 11, 13, and 15, and the corresponding sensitivity test results are shown in Figure 4.

### 3.2. Different Methods of Segmentation and Comparison

**Figure 5:**
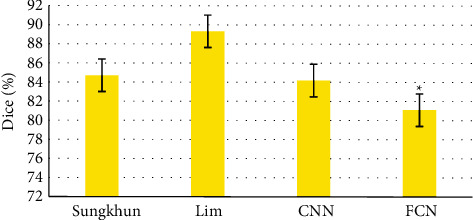
Comparison of segmentation results of different methods.

**Figure 6:**
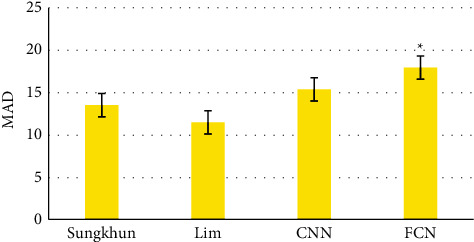
Comparison of segmentation mean absolute distance results for different methods. *∗*Compared with other groups, *P* < 0.05.

The segmentation results of different methods in references [20, 21], FCN, and CNN were compared. The results showed that the segmentation speed of FCN improved by CNN was significantly improved, and the average absolute distance of FCN was significantly better than the other three methods (*P* < 0.05, Figures 5 and 6).

### 3.3. Image Processing Results of Patients with AIS

**Figure 7:**
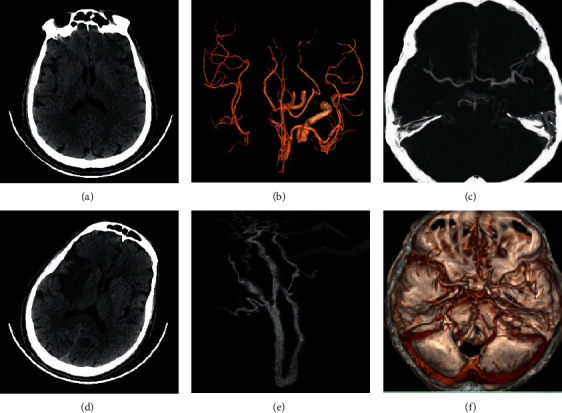
The image of the case.

Figure 7 shows a 65-year-old patient who had intermittent dizziness for more than 5 years, with good compensation, and was followed up for observation in the later period. Figures 7(a) and 7(b) are CT images. After deep learning processing, the image was clear, and the cerebral perfusion imaging arteries were clearly visible. Figures 7(c)–7(f) are all cerebral artery imaging results. After being processed by intelligent algorithm, the images were clearly visible and the veins of blood vessels were clear.

**Figure 8:**
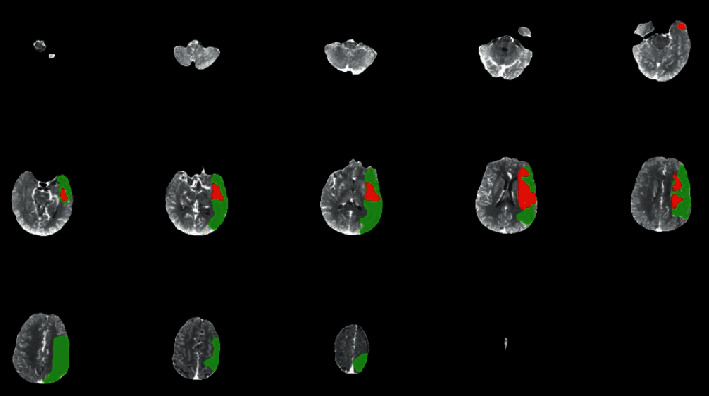
The segmented images.

For 4D CTA and whole brain CTP imaging, the CNN-based network showed good results for image segmentation. Postoperatively, whole brain perfusion imaging showed hypoperfusion changes in the cerebral arterial supply area, and the segmented region of interest showed a good degree of accuracy (Figure 8).

### 3.4. Comparison of Patient Imaging-Related Indicators

**Figure 9:**
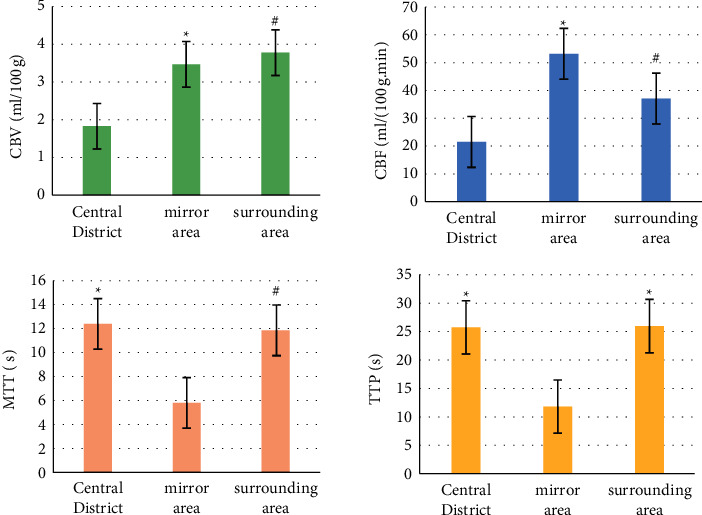
Comparison of parameters in different areas of perfusion imaging. *∗* and # indicated that the difference was significant, *P* < 0.05.

The parameters of the central area, the peripheral area, and the mirror area of the perfusion map were compared, and the results are shown in Figure 9. Compared with the mirror area, the MTT and TTP of the lesion were prolonged, the peripheral CBV was increased, and the differences between the parameters were significant (*P* < 0.05).

## 4. Discussion

Stroke is about 10% of brain diseases, including acute ischemic cerebral hemorrhage disease and acute hemorrhagic cerebrovascular disease. In recent years, the incidence of stroke shows a trend of younger age. How to make the early diagnosis of acute cerebral infarction, determine the ischemic penumbra, actively treat thrombolysis within the effective reperfusion time, and implement effective brain protection measures have been the hot spots of acute cerebral infarction research in recent years [22, 23]. Hypoperfusion is the final pathway of all etiological mechanisms of cerebral ischemia. Dynamic CTP imaging can clearly display hemodynamic abnormalities in the early stage of cerebral infarction and provide relevant functional information of cerebral hemodynamics according to the ratio and correlation of various parameters [24]. In this study, CBV in peripheral area increased, while CBV and CBF in central area decreased. Zhang et al. [25] performed image analysis of anterior circulation patients with AIS, including one-stop whole brain dynamic volume four-dimensional CTA and cranial imaging for all patients. CT angiography parameters of patients have important value for the treatment prognosis of patients with AIS. CTP-ASPECTS scores were negatively correlated with clinical prognosis. The higher the CTP-ASPECTS score of patients with anterior circulation ischemic stroke before treatment, the better the prognosis. 4D CTA combined with CTP imaging feature analysis can provide changes in cerebral vascular morphology in cerebral stroke. Wagemans et al. [26] showed that 4D CTA improved the diagnostic accuracy of proximal intracranial anterior circulation occlusion in acute stroke. 4D CTA, used as an additive to conventional CTA and CTP in patients with AIS eligible for intraarterial therapy, showed a tendency to increase diagnostic accuracy and improve diagnostic certainty when reviewed by radiologists in training, while only slightly extending the time to diagnosis. Craniocerebral CT plain scan and CTP imaging for patients with suspected ischemic cerebrovascular disease can be comprehensively evaluated from multiple perspectives, to identify responsible lesions and blood vessels, providing objective, comprehensive, and reliable image basis for physicians to make rational clinical decisions. However, the disadvantages are radiation dose and high examination cost. Cao et al. [9] used 4D CTA and a comprehensive and objective scoring system to evaluate collateral circulation, and 4D CTA can be used to effectively evaluate the status of collateral circulation. Accurate assessment of collateral circulation based on 4D CTA will help make medical decisions, especially for patients who will undergo endovascular intervention. The results showed that 4D CTA combined with CTP imaging based on deep learning had important clinical application value.

In this study, 4D CTA and whole brain CTP imaging based on FCN segmentation algorithm were used to examine patients with AIS, and abnormal cerebral blood flow could be found. Each parameter can be used to quantitatively evaluate the degree of ischemic damage in brain tissue. CTP imaging technology optimized by FCN can be used to analyze cerebral perfusion status. However, there was still no unified standard for individual differential memory perfusion conditions, and there was no corresponding consensus on the optimal perfusion threshold of penumbra and cerebral infarction area. Compared with the mirror area, the MTT and TTP of the lesions were prolonged, the peripheral CBV was increased, and the parameters were significantly different (*P* < 0.05). The image segmentation based on FCN showed good results.

## 5. Conclusion

In this study, 4D CTA was combined with CTP imaging based on deep learning to explore the changes of cerebrovascular morphology and hemodynamics. Deep learning intelligent segmentation algorithm applied to AIS CTP imaging can effectively improve patient image features and improve classification efficiency. Nevertheless, there are still some limitations in this study. The interference of subjective factors cannot be excluded from the experimental data, and specific standardization of each indicator is required in future research.

## Figures and Tables

**Table 1:** CTP findings before cerebral infarction.

Stage	CTP manifestations	Microcirculation state	Cerebral blood flow status
I_1_	All parameters were normal	Well-established local collateral circulation in the brain	No drop in CBF
I_2_	Prolonged TTP and normal MTTCBF and CBV	Uncompensated dilation of microvessels in the brain	CBF above electrical failure threshold
I_3_	Prolonged TTP and MTT, normal/slightly decreased CBF, and elevated CBV	Compensatory dilation of microvessels in the brain	CBF above electrical failure threshold
II_1_	Prolonged TTP and MTT, decreased CBF, and normal/slightly decreased CBV	Mild stenosis of local microvascular compression in the brain	CBF between electrical failure threshold and membrane failure
II_2_	Prolonged TTP and MTT and decreased CBF and CBV	The microvessels in the brain were obviously compressed and narrowed	CBF between electrical failure threshold and membrane failure

## Data Availability

The data used to support the findings of this study are available from the corresponding author upon request.
